# The Blood-Pressure-Lowering Effect of Food-Protein-Derived Peptides: A Meta-Analysis of Recent Clinical Trials

**DOI:** 10.3390/foods10102316

**Published:** 2021-09-29

**Authors:** Wang Liao, Guiju Sun, Dengfeng Xu, Yuanyuan Wang, Yifei Lu, Jihan Sun, Hui Xia, Shaokang Wang

**Affiliations:** Key Laboratory of Environmental Medicine and Engineering of Ministry of Education, and Department of Nutrition and Food Hygiene, School of Public Health, Southeast University, Nanjing 210009, China; wangliao@seu.edu.cn (W.L.); withxu@seu.edu.cn (D.X.); 230218460@seu.edu.cn (Y.W.); 230198332@seu.edu.cn (Y.L.); 230198876@seu.edu.cn (J.S.); huixia@seu.edu.cn (H.X.); shaokangwang@seu.edu.cn (S.W.)

**Keywords:** food-protein-derived peptides, blood pressure, meta-analysis

## Abstract

Although clinical trials of food-protein-derived peptides in the management of hypertension have been published, the results are controversial, which compelled us to conduct a meta-analysis to evaluate the pooled effect of peptide intervention. In this study, we searched for studies published between 2010 and 2021 and selected 12 eligible studies for a meta-analysis. The pooled effect of peptide intervention for systolic blood pressure (SBP) and diastolic blood pressure (DBP) was −3.28 mmHg (95% CI: −4.54, −2.03, *p* < 0.001) and −1.82 mmHg (95% CI: −3.46, −0.18, *p* = 0.03), respectively. Sub-group analyses showed that the reduction in BP in participants with higher basal BP (>140/85 mmHg) was greater (*p* = 0.007 for SBP and *p* = 0.01 for DBP), and the effect was stronger in Asian participants as compared with non-Asian participants (*p* = 0.01 for SBP and *p* = 0.04 for DBP). In addition, the effect of peptide intervention was more pronounced on SBP in participant groups with a lower ratio of male to female (≤0.5) as well as in participants with a mean age ≥50 years old. In conclusion, food-protein-derived antihypertensive peptides can significantly reduce BP in prehypertensive and hypertensive patients. Findings from this study could provide guidance for the design of clinical trials of antihypertensive peptides.

## 1. Introduction

Hypertension ranks as the top cause of cardiovascular disease. Although the global mean blood pressure has remained constant in recent decades due to antihypertensive medications, the prevalence of hypertension has continued to increase [1]. In addition, an upward trend of hypertension in young populations has also been observed over the past two decades [2]. Thus, hypertension is considered a global health challenge. As cardiovascular diseases, caused by hypertension, are the leading contributor to mortality worldwide [3], the cost of healthcare associated with hypertension and other complications has become a social economic burden. In addition, the prolonged use of synthetic antihypertensive drugs always has side-effects. Therefore, it is necessary to develop novel strategies with fewer adverse effects and lower costs to manage hypertension. There is a consensus that controlling high blood pressure (BP) using dietary and natural products is highly encouraged. Phytochemicals including soy isoflavones and resveratrol are typical examples within the recommended class of natural products [4,5]. However, the availability of other natural products, in addition to phytochemicals, that contain strong clinical evidence in reducing BP is an open question.

Bioactive peptides are oligopeptides liberated from food proteins, which can exert physiological activities in addition to their nutritional values. Activities of the peptides are present once they are produced from their parent proteins by hydrolysis or fermentation. As bioactive peptides are naturally derived, they are considered promising alternatives for the management of chronic diseases, including hypertension [6]. Various peptides with the potential for antihypertensive activity have been characterized from animal- or plant-based food protein sources [7]. Previously, an abundance of work on antihypertensive peptides has concentrated on protein hydrolysate preparation, peptide identification, animal work-based activity evaluation, and mechanistic studies [8].

Although clinical evidence of food-protein-derived peptides in reducing BP has been reported, most of the previous clinical studies of antihypertensive peptides evaluated the effect of milk-protein-derived peptides, and related meta-analyses have already been published [9,10]. Notably, clinical trials of antihypertensive peptides derived from food proteins other than milk proteins have been published in the last decade [11]. However, most of these clinical studies are randomized clinical trials (RCTs), recruiting specified participants. The outcomes of some studies were controversial [12]. A comprehensive overview of the antihypertensive activity of bioactive peptides in humans was found to be lacking, which has been a major factor impeding the commercialization of antihypertensive peptides. Since antihypertensive peptides have been identified from a number of food proteins, it is necessary to carry out a comprehensive review on the recent research progress of clinical trials of antihypertensive peptides and conduct a meta-analysis.

It is necessary to further investigate the effect of antihypertensive peptides and identify the key factors that may affect their effect size. Thus, we carried out a quantitative synthesis of evidence since 2010 and conducted a meta-analysis to assess the effect of antihypertensive peptides in humans.

## 2. Materials and Methods

This meta-analysis followed the recommendations of the preferred reporting items for systematic reviews and meta-analyses (PRISMA) statement [13].

### 2.1. Search Strategy and the Inclusion and Exclusion Criteria

Database including PubMed and Web of Science were used to search for clinical trials investigating the antihypertensive effects of food-protein-derived bioactive peptides published between 2010 and up to 15 July 2021. The search was performed using the following strings: “Bioactive peptides” OR “Hydrolysate” AND “Blood pressure” OR “Hypertension” AND “Trial”. In addition, potentially eligible studies from review articles were manually searched.

The selection of studies to be included in this meta-analysis was based on the following eligibility criteria: RCT or a cross-over study that assessed the effects of bioactive peptides on blood pressure in adults (aged 18 or above); the primary outcome measurement was data from either office blood pressure measurement or ambulatory blood pressure monitoring; the subjects should be defined as “prehypertension” or “hypertension” according to the most recent guidelines [14] and the intervention period should be at least 1 week. The retrieved studies were screened based on the title and abstract. Trials excluded in the screening step included studies that used animal models; studies that evaluated the effect of an intact food protein or single amino acid instead of a peptide; studies that investigated acute effects instead of chronic effects of the peptide; and studies that were not relevant to the scope of this meta-analysis. After the first step of screening, the full texts of the remaining studies were reviewed to determine if they were eligible to be included in this meta-analysis following the criteria mentioned above. All of the studies were reviewed by two investigators independently. When there was disagreement, a third reviewer joined the discussion until an agreement was reached.

### 2.2. Data Extraction

The title and abstract of each piece of literature was screened to determine whether the trial was eligible to be included. The following information of the included studies was extracted: authors; publication year; country of the study; study design; protein source of the peptide; intervention dosage; intervention duration; mean age of the subjects; the ratio of male to female of the participants; basal systolic/diastolic BP (SBP/DBP); change of systolic/diastolic pressure and the approach to BP measurement. The treatment effect was defined as the mean difference in BP change between the active and control groups. For trials with the cross-over design, the first-period data were collected to avoid disturbance from the wash-out period. The information from the highest dose group was used when there was more than one dose involved.

### 2.3. Statistical Analyses

The random effect model was applied to measure the difference of the effect size. The effect size of the treatment was calculated by subtracting the basal SBP or DBP from the corresponding SBP or DBP at the end point. The sub-group analyses were also conducted by using the random effect model, which included analyses of the effects of basal BP on the participants, the age of the participants, the ratio of male to female in each trial, the trial size, the duration of the treatment, the delivery vehicle of the peptides, and the protein source of the peptides. All of the analyses were run with a 5% level of significance. All of the data analyses were run via Review Manager 5.4 and Stat 11.0 software.

The heterogeneity was evaluated via the I^2^ statistics. An I^2^ > 75% was considered as a high level of heterogeneity. Furthermore, the publication bias was evaluated by a funnel plot and Egger’s test. The quality assessment of each individual study was examined based on the Cochrane risk of bias tool [15]. Each study was evaluated according to each item and scored as a high, unclear, or low risk of bias.

## 3. Results

### 3.1. Study Characteristics

**Figure 1:**
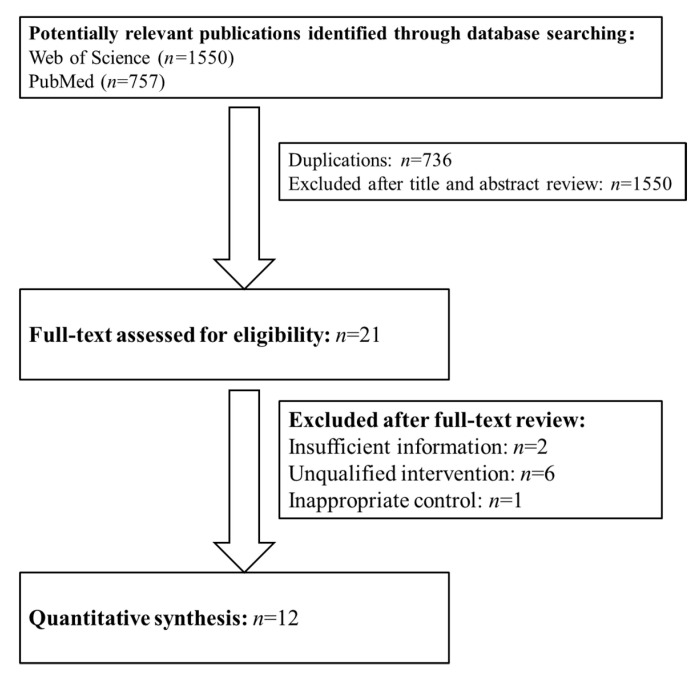
The flow diagram of trial selection.

In total, 2307 potentially relevant publications were identified. After removing the duplications (*n* = 736) and reviewing the titles and abstracts, we excluded 2286 studies. The full texts of the remaining 21 publications were reviewed, and 12 studies were included in this meta-analysis according to the inclusion and exclusion criteria (Figure 1).

With regard to the included studies, all 12 of these studies were published in English in peer-reviewed journals as original research articles (Table 1). In total, the 12 reported trials included 761 participants. All of the included studies were randomized and double blind. For the intervention of each study, milk-protein-derived peptides were used in eight studies [16,17,18,19,20,21,22,23], egg protein-derived bioactive peptides were used in two trials [24,25], and a chicken collagen-derived peptide was used in one study [26]. Only one study used plant-derived peptides that were from rice bran [27]. Although there were two studies that investigated the blood-pressure-lowering effect of legume-derived peptides in humans, these trials were excluded due to the insufficient information of the participants [28] or the fact that the treatment was also combined with other nature compounds [29]. From the primary literature, there was one trial that evaluated the effect of marine collagen-derived peptides, while the recruited participants were patients with type 2 diabetes as well as hypertension [30]. Therefore, the reduction in blood pressure after the peptide intervention might be due to the mitigation of type 2 diabetes instead of the direct effect on blood pressure. Thus, this study was also excluded. Protein hydrolysis and fermentation are major approaches for the production of bioactive peptides in large scale. For the 12 included studies, 8 of them used protein hydrolysates and 4 used peptides via fermentation.

### 3.2. The Effects of Bioactive Peptide Intervention

**Figure 2:**
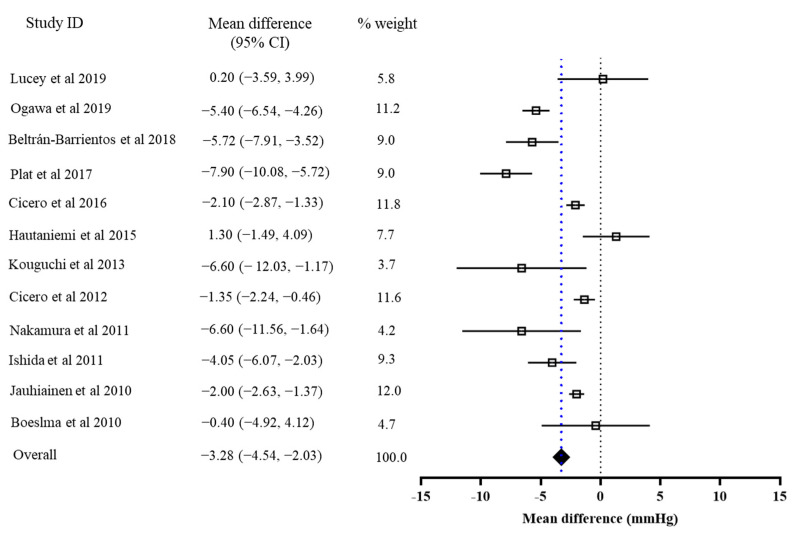
Overall change in SBP (mmHg) after peptide intervention.

**Figure 3:**
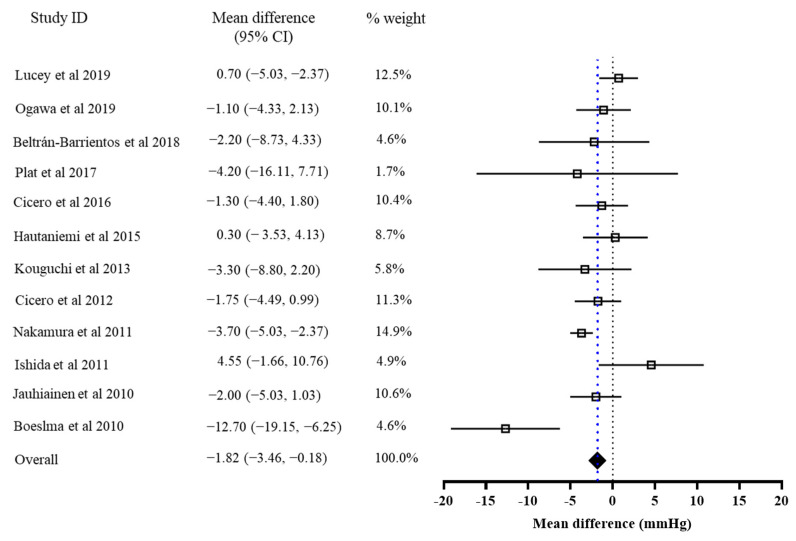
Overall change in DBP (mmHg) after the peptide intervention.

The results of the primary meta-analysis showed that the intervention of bioactive peptides reduced SBP and DBP by 3.28 mmHg (95% CI: −4.54, −2.03, *p* < 0.001) and 1.82 mmHg (95% CI: −3.46, −0.18, *p* = 0.03), respectively. This result suggested a significant effect of the intervention of bioactive peptides on the reductions in both SBP and DBP. Both of the pooled effects for SBP (I2 = 70%, Tau^2^ = 0.17, Chi^2^ = 37.20, df = 11, *p* = 0.0001) and DBP (I2 = 55%, Tau2 = 0.80, Chi2 = 24.49, df = 11, *p* = 0.003) were heterogeneous (Figure 2 and Figure 3).

We then conducted sub-group analyses. As shown by Table 2, it was found that the basal BP of the participants affected the effect sizes of both SBP (*p* = 0.007) and DBP (*p* = 001). Higher basal BP (>140/85 mmHg) was associated with a greater effect size (Table 2). The ratio of male to female of the participants affected the effect size of SBP significantly (*p* = 0.04), in which a lower ratio (≤0.5) indicated a more pronounced effect size. A similar trend was also present in the effect size of DBP, although the effect did not have any statistical significance (*p* = 0.06). In line with a previous study, we found that the Asian participants had a stronger response to the peptide intervention (SBP: *p* = 0.01 DBP: *p* = 0.04)as compared with the participants from other countries (seven studies recruited participants from European countries and one study recruited a participant from Mexico). We also found that the peptide intervention in the participants with a mean age above 50 years old had a more pronounced effect on the reduction in SBP (*p* = 0.001) but not the reduction in DBP (*p* = 0.88). In fact, the trial size could significantly affect the effect size of DBP (*p* = 0.04) but not SBP (*p* = 0.35). Most of the included trials used milk-protein-derived peptides. However, the origin of the peptide might not significantly affect the effect size of the intervention (SBP: *p* = 0.07, DBP: *p* = 0.20). In addition, neither the duration nor the delivery vehicle of peptides affected the effect size of the peptide intervention.

### 3.3. Publication Bias

**Figure 4:**
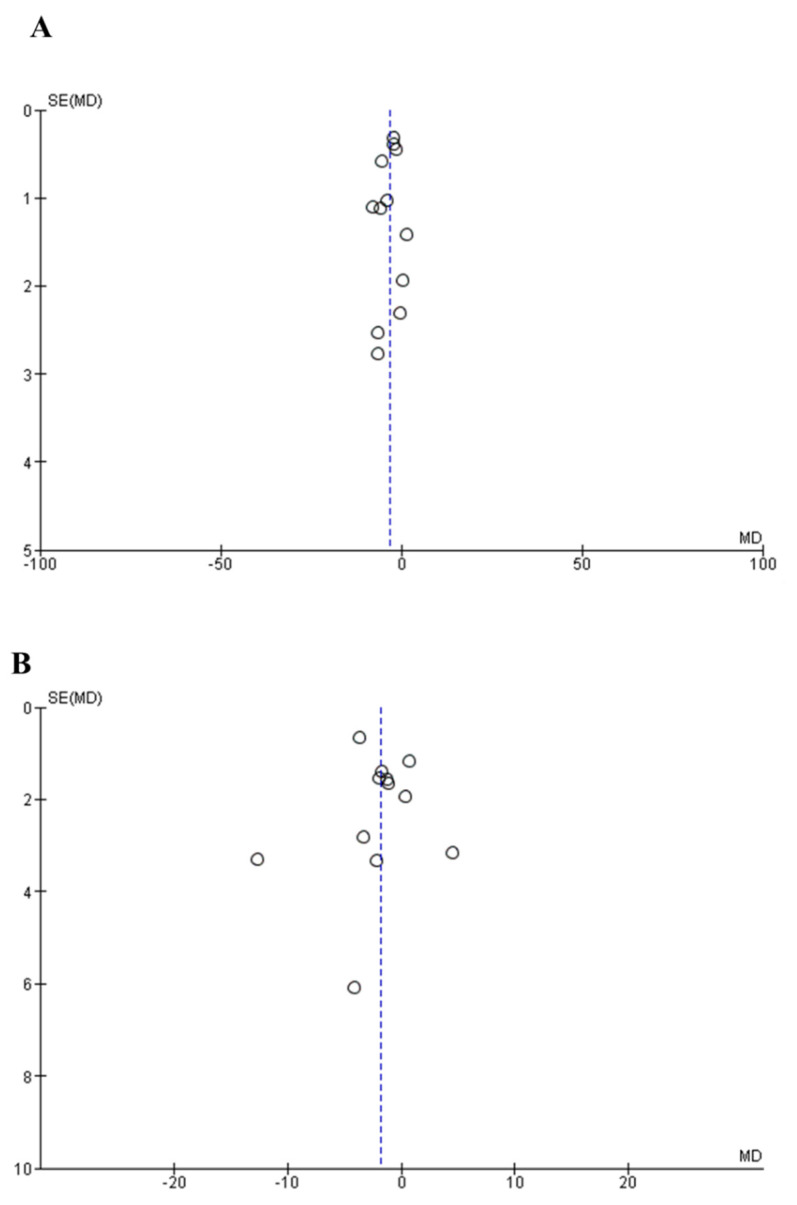
Funnel plot measuring publication bias and the effects of peptide intervention on SBP (**A**) and DBP (**B**). The X axis represents mean difference (MD) and the Y axis represents standard error (SE) of the MD.

The publication bias was evaluated using the funnel plot and Egger’s test. There was no visual asymmetry in the funnel plots (Figure 4A,B). The *p* values of Egger’s tests for SBP and DBP were 0.43 and 0.79, respectively. Collectively, the above results indicated that publication bias existed in the trials involved in this analysis.

We then assessed the risk of bias of each included study based on the Cochrane guidelines. As shown in Table 3, a high risk was present in the blinding of both participants and personnel, as well as in the creation of incomplete outcome data of some studies, which indicated selection bias, performance bias, and attrition bias, respectively. In addition, the bias of random sequence generation and allocation concealment in most of the included studies were unknown, which suggested a potential selection bias in these trials.

## 4. Discussion

Bioactive peptides from food proteins have attracted enormous attention in the past few years due to their potential use in the management of chronic metabolic diseases, including hypertension. Since the first identification of an antihypertensive peptide from snake venom four decades ago, great efforts have been placed on the identification of more antihypertensive peptides as well as on cellular and animal studies to evaluate their activities and mechanisms [6]. The activity of antihypertensive peptides from milk, egg, and chicken proteins has also been investigated. However, the effects of these peptides on hypertensive subjects are controversial [11,12]. Thus, collection of evidence and meta-analyses are warranted to generate a comprehensive view on the activity of antihypertensive peptides.

To date, most clinical trials of antihypertensive peptides have concentrated on lactopeptides, and several meta-analyses that assessed the effects of these lactopeptides have been published [9,10,31]. Notably, clinical trials of antihypertensive peptides derived from proteins other than milk proteins have appeared in recent years. Therefore, we included the qualified clinical trials published since 2010 concerning the antihypertensive peptides derived from various food protein sources and conducted a meta-analysis in the present study. Although the number of total participants involved in this meta-analysis was not as large as previous meta-analyses [9,10,31], this meta-analysis is important to the relevant field since we included the most recent trials of peptides derived from various food protein sources instead of milk proteins only. We found that peptides derived from egg, chicken collagen, and rice proteins showed a comparable BP-lowering effect to the effect size of milk-protein-derived peptides. Therefore, it will be helpful to explore the BP-lowering activity of peptides originating from different types of protein sources in future clinical trials.

In this study, we found that antihypertensive peptides exerted a significant effect in reducing both SBP and DBP. The intervention of antihypertensive peptides was indicated to reduce the body weight of hypertensive patients by some studies [18,20], but such an effect is still ambiguous. As such, it will be helpful to collect more types of evidence, such as body weight and blood lipid profiles, in future clinical trials, which may be helpful in unveiling the mechanisms of peptides underlying the antihypertensive activity in humans.

The results from sub-group analyses suggested that the basal BP of the participants could be a key factor affecting the effect size. The effect of peptide intervention in participants with a higher basal BP (SBP > 140 mmHg and DBP < 85 mmHg) was more pronounced than in participants with a lower basal BP, which is consistent with a previous study that reported the effect of lactotripeptides was stronger in hypertensive subjects than in non-hypertensive subjects [32]. This finding also suggested that food protein-derived antihypertensive peptides may reduce BP by normalizing the dysregulated metabolic system.

Surprisingly, we found in the present meta-analysis that the ratio of male to female also affected the effect size of the peptide intervention. The peptide intervention had a stronger effect on SBP in participants with the ratio of male to female being less than 0.50. Similar trends also existed in the effect of peptides on DBP. Such findings indicated that antihypertensive peptides may favor female subjects over their male counterparts. Differential regulatory roles of bioactive compounds in males and females have attracted a considerable amount of attention in recent years [33,34]. Previous research of food-protein-derived bioactive peptides did not consider this important point. For antihypertensive peptides in particular, as the prevalence of hypertension in males is higher than in age-matched females before menopause, spontaneously hypertensive male rats were used as the model animal in most of the animal studies. In clinical trials of antihypertensive peptides, the data of male and female participants were always pooled together. Since we found that males and females might have different responses to peptide intervention, future clinical trials of antihypertensive peptides are encouraged to separate different genders and explore whether the effects and underlying mechanisms of peptides are different.

In this meta-analysis, we also found that the effect size of the peptide intervention could be affected by the age of the participant, which was a factor ignored in previous meta-analyses of antihypertensive peptides. Interestingly, peptide intervention had a significantly stronger effect on SBP in subjects with the mean age > 50 years. This finding may explain a previous study reporting the non-significant effect of lactotripeptides in hypertensive patients, in which the age of the recruited participants ranged from 35 to 70 years old [12]. Such a broad age range might mask the effect of peptides on the younger group. However, only four trials with an average age ≤50 were collected in this meta-analysis. More evidence is required to demonstrate that antihypertensive peptides have a better effect on older patients.

Although sub-group analyses involved the intervention duration and delivery vehicle of the peptides, neither of these two factors had a significant effect on the change in BP. Future trials are suggested to contain designs with a wider dose range and different intervention periods. In addition, the molecular mechanisms of a food-protein-derived bioactive peptide underlying its blood-pressure-lowering effect have been explored, which included inhibition of the angiotensin-converting enzyme activity to reduce the concentration of vasoconstrictor angiotensin II, activation of the angiotensin converting enzyme 2 activity to mitigate vascular inflammation and oxidative stress, and activation of the endothelial nitric oxide-signaling to enhance vascular relaxation [6,7,11]. However, as these mechanistic studies were conducted on animal models, we suggest further exploring the blood-pressure-reducing mechanisms in humans in future trials.

## 5. Conclusions

In conclusion, food-protein-derived antihypertensive peptides can reduce BP in hypertensive individuals significantly. The basal BP, age, and gender of the participants may alter the outcome of the intervention. The findings of this meta-analysis shed light on the factors that may impact the activity of antihypertensive peptides in humans. In addition, the findings of this study could provide guidance for the design of clinical trials of antihypertensive peptides. It must be admitted that publication bias is present in the studies involved in this meta-analysis, which compels us to conduct more clinical trials of antihypertensive peptides.

## Figures and Tables

**Table 1:** Characteristics of the included trials.

Study	Number of Participants (M/F), Average Age	Study Design	Duration	Treatment	Daily Dosage	Placebo	Basal BP		BP Measurement
							Systolic BP	Diastolic BP	
Jauhiainen et al., 2010 [16]	121 (54/67), 49 ± 5	R, D, PAL, P	12 weeks	IPP and VPP in fermented milk	50 mg IPP and VPP	Placebo milk without peptides	T: 151.3 ± 14.8 C: 154.6 ± 13.9	T: 95.2 ± 12.2 C: 94.2 ± 8.8	ABPM
Boeslma et al., 2010 [17]	26 (17/9), 59 ± 7.3	R, D, PAL, P, CR	4 weeks	Casein hydrolysate	1 g hydrolysate in 2 capsules	2 capsules filled with cellulose	T: 146.6 ± 10.8 C: 150.4 ± 8.9	T: 89.7 ± 8.7 C: 92.0 ± 8.5	Office
Ishida et al., 2011 [18]	32 (16/16), 51.9 ± 9.3	R, D, PAL, P	6 weeks	IPP and VPP in casein hydrolysate	7.5 mg VPP and 9.6 mg IPP in tablets	Tablets filled with sodium caseinate	T: 141.4 ± 3.5 C: 141.3 ± 3	T: 84.7 ± 5.1 C: 86.1 ± 5.1	Office
Nakamura et al., 2011 [19]	70 (47/23), 57.8 ± 5.4	R, D, PAL, P	8 weeks	IPP and VPP in casein hydrolysate	1.5 mg VPP/day and 1.9 mg IPP in tablets	Tablets filled with sodium caseinate	T: 146.8 ± 4.4 C: 87.5 ± 7.1	T: 146.9 ± 4.3 C: 88.0 ± 7.7	Office
Cicero et al., 2012 [20]	164 (101/63), 43.85 ± 11.1	R, D, PAL, P	4 weeks	IPP and VPP in milk protein hydrolysate	2 mg VPP/day and 1 mg IPP with 250 mL fruit juice	250 mL fruit juice without peptides	T: 133.49 ± 12.92 C: 83.28 ± 8.75	T: 132.69 ± 12.46 C: 82.78 ± 8.33	ABPM
Kouguchi et al., 2013 [26]	58 (30/28), 52.8 ± 8.5	R, D, PAL, P	12 weeks	Chicken collagen hydrolysate	4.4 g hydrolysate in 120 mL of lactic acid drink	120 mL of lactic acid drink without hydrolysate	T: 139.2 ± 9.1 C: 85 ± 7.6	T: 137.9 ± 11.1 C: 85.5 ± 7.4	Office
Hautaniemi et al., 2015 [19]	58 (30/28), 52.3 ± 6.6	R, D, PAL, P	12 weeks	IPP and VPP in fermented milk	50 mg IPP and VPP in 125 mL fermented milk	125 mL fermented milk without peptides	T: 168 ± 20 C: 162 ± 15	T: 102 ± 10 C: 100 ± 10	Office
Cicero et al., 2016 [21]	40 (26/14), 50.1 ± 9.9	R, D, PAL, P, CR	4 weeks	IPP and VPP in casein hydrolysate	10.2 mg IPP and VPP in 12 tablets	12 tablets filled without peptides	T: 142.1 ± 11.5 C: 139.2 ± 8.3	T: 86.2 ± 8.6 C: 85.2 ± 7.2	ABPM
Plat et al., 2017 [24]	10	R, D, PAL, P, CR	1 week	Egg protein hydrolysate	2 g hydrolysate	Erythritol	T: 148.1 ± 12.9 C: 143.6 ± 9.3	T: 87.9 ± 10.0 C: 85.3 ± 9.2	Office
Beltrán-Barrientos et al., 2018 [23]	36 (20/16), 42.55 ± 10.45	R, D, PAL, P	5 weeks	Fermented milk	150 mL	Control milk	T: 131.8 ± 5.6 C: 134.3 ± 7	T: 87.7 ± 5.1 C: 89.3 ± 5.8	Office
Lucey et al., 2019 [25]	65 (37/28), 56.9 ± 5.2	R, D, PAL, P, CR	12 weeks	Ovalbumin hydrolysate	3 g hydrolysate in 150 mL fruit juice	Maltodextrin in 150 mL fruit juice	T: 135.5 ± 12.3 C: 133.8 ± 10.6	T: 75.9 ± 6.3 C: 76.1 ± 7	Office
Ogawa et al., 2019 [27]	71 (31/40), 53.9 ± 5.85	R, D, PAL, P	12 weeks	Rice bran peptide LRA	43 μg peptide in 4 tablets	4 tablets without peptides	T: 141 ± 8.5 C: 141.9 ± 8.5	T: 89.4 ± 7 C: 88.9 ± 6.4	Office

BP: Blood pressure; R: Randomized; D: Double blind; PAL: Parallel; P: Placebo-controlled; CR: Crossover; ABPM: Ambulatory blood pressure monitoring; T: Treatment; C: Control.

**Table 2:** Sub-group analyses of the included trials.

Sub-Group Title	No. of Trials	Mean Difference (95% ci)	
		SBP	DBP
Basal BP			
≤140/85 mmHg	4	−1.97 (−2.77, −1.18)	−0.25 (−2.67, 2.17)
>140/85 mmHg	8	−2.74 (−3.16, −2.32)	−2.69 (−3.69, −1.69)
*p* value		0.007	0.01
Ratio of male to female			
≤0.5	6	−4.29 (−6.39, −2.19)	−0.36 (−1.92, 1.20)
>0.5	6	−1.75 (−3.08, −0.42)	−2.96 (−5.20, −0.71)
*p* value		0.04	0.06
Age			
≤50	4	−2.02 (−2.44, −1.60)	−1.73 (−3.37, −0.09)
>50	8	−4.62 (−5.42, −3.81)	−1.96 (−4.55, 0.63)
*p* value		0.001	0.88
Country of the study			
Asia (Japan)	4	−1.19 (−1.45, −0.94)	−3.04 (−4.22, −1.87)
Others (Europe and Mexico)	8	−0.45 (−0.64, −0.26)	−2.18 (−3.03, −1.33)
*p* value		0.01	0.04
Trial size			
≤50	5	−4.06 (−6.54, −1.57)	−0.10 (−1.59, 1.79)
>50	7	−2.62 (−4.33, −0.91)	−2.68 (−4.74, −0.63)
*p* value		0.35	0.04
Parent protein			
Milk protein	8	−2.37 (−3.38, −1.36)	−2.13 (−4.22, −0.04)
Other protein sources	4	−5.11 (−7.89, −2.34)	−0.33 (−2.09, 1.42)
*p* value		0.07	0.20
Intervention duration			
≤6 weeks	6	−3.67 (−5.49, −1.84)	−2.53 (−6.16, 1.09)
>6 weeks	6	−2.86 (−5.17, −0.55)	−1.38 (−2.92, 0.15)
*p* value		0.59	0.57
Delivery vehicle			
Liquid	6	−2.07 (−3.53, −0.61)	−0.82 (−2.16, 0.52)
Non-liquid	6	−4.46 (−6.60, −2.32)	−2.70 (−5.69, 0.30)
*p* value		0.07	0.26

**Table 3:** Quality assessments of included studies based on the Cochrane guidelines.

Study	Random Sequence Generation	Allocation Concealment	Blinding of Participants and Personnel	Blinding of Outcome Assessment	Incomplete Outcome Data	Selective Reporting	Other Sources of Bias
Jauhiainen et al., 2010 [16]	U	U	L	L	L	L	L
Boeslma et al., 2010 [17]	U	U	L	L	H	L	L
Ishida et al., 2011 [18]	U	U	L	U	L	L	L
Nakamura et al., 2011 [19]	U	U	L	U	L	L	L
Cicero et al., 2012 [20]	U	U	L	L	L	L	L
Kouguchi et al., 2013 [26]	U	U	L	U	L	L	L
Hautaniemi et al., 2015 [21]	U	U	L	U	L	L	L
Cicero et al., 2016 [22]	U	U	L	L	L	L	L
Plat et al., 2017 [24]	U	H	H	U	H	L	L
Beltrán-Barrientos et al., 2018 [23]	U	U	L	U	L	L	L
Lucey et al., 2019 [25]	U	U	L	U	H	L	L
Ogawa et al., 2019 [27]	U	U	L	U	L	L	L

U: Unknown; L: Low risk; H: High risk.
